# Trends in the age-standardized prevalence of neurodevelopmental disorders in South America, 1990–2023

**DOI:** 10.1055/s-0046-1820532

**Published:** 2026-05-12

**Authors:** Mauricio Lopez-Espejo

**Affiliations:** 1 Pontificia Universidad Católica de Chile, Escuela de Medicina, División de Pediatría, Sección de Neurología Pediátrica, Santiago, Chile.; 2 Red Salud UC CHRISTUS, Unidad de Neurodesarrollo, Santiago, Chile.

**Keywords:** Autism Spectrum Disorder, Attention Deficit Disorder with Hyperactivity, Intellectual Disability, Global Burden of Disease, Epidemiology, Prevalence, South America, Sex Differences

## Abstract

**Background:**

Neurodevelopmental disorders (NDDs) are substantial burdens for individuals and health systems; yet, their long-term epidemiology in South America is poorly characterized.

**Objective:**

To describe the trends from 1990 to 2023 in the age-standardized prevalence rates (ASPRs) of autism spectrum disorder (ASD), attention-deficit/hyperactivity disorder (ADHD), and idiopathic developmental intellectual disability (IDID) across 12 South American countries using Global Burden of Disease (GBD) 2023 estimates.

**Methods:**

Country-specific annual ASPRs and 95% uncertainty intervals (95%UIs) were obtained from the GBD 2023. Sex-stratified ASPRs were used to calculate male-to-female ratios (MFRs) and summarize long-term sex disparities. Temporal trends were assessed using the average annual percent change (AAPC) from log-linear regression for each country.

**Results:**

In 2023, the ASPRs ranged from 0.57 to 1.06% for ASD, from 1.00 to 2.55% for ADHD, and from 0.42 to 0.54% for IDID across countries. Between 1990 and 2023, the ASD ASPRs increased in all countries, with AAPCs from +0.35%/year (Paraguay) to +0.79%/year (Chile). The ADHD ASPRs showed little net change, with AAPCs from −0.10%/year (Argentina) to +0.39%/year (Brazil). And the IDID ASPRs declined consistently, with AAPCs from −0.79%/year (Bolivia) to −0.30%/year (Suriname). Sex differences in ASPRs were large and persistent for ASD and ADHD, but small for IDID. The MFR trajectories suggested a slight widening of the ASD male–female gap, largely stable ADHD disparities, and minimal variability for IDID.

**Conclusion:**

Over 3 decades, the ASD ASPRs rose modestly, the ADHD ASPRs were broadly stable, and the IDID ASPRs declined across South America. The GBD 2023 estimates point to enduring diagnostic and structural inequities and to the need for strengthened surveillance and more equitable access to developmental assessment.

## INTRODUCTION

Neurodevelopmental disorders (NDDs) comprise a broad group of conditions arising from early alterations in brain development. They can impair learning, communication, adaptive functioning, and behavior across the life course, with consequences that extend beyond the affected individuals to families, health systems, and societies through long-term support needs, reduced productivity, and unequal access to specialized services. 1 Although symptoms often first appear in childhood, an increasing number of individuals receive an initial diagnosis in adolescence or adulthood. 2 3

Among the most frequently studied NDDs are *autism spectrum disorder* (ASD), characterized by persistent difficulties in social communication and interaction, with restricted and repetitive behaviors; *attention-deficit/hyperactivity disorder* (ADHD), defined by impairing patterns of inattention and/or hyperactivity–impulsivity; and *idiopathic developmental intellectual disability* (IDID), a subtype of intellectual disability marked by significant limitations in cognitive and adaptive functioning without a known etiology, with onset during the developmental period. 4 5

According to the Global Burden of Disease 2021 (GBD 2021) study, the global age-standardized prevalence rates (ASPRs) in 2021 were 1.11% for ADHD, 0.79% for ASD, and 1.16% for IDID. 6 7 Over the preceding 3 decades, the global ASPRs showed modest increases for ASD and declines for ADHD and IDID. 6 7 8 9 10 11 These patterns are thought to reflect evolving diagnostic practices, public awareness, and risk-factor profiles, along with wider sociocultural and environmental influences.

In South America, longstanding inequities in health-system capacity, economic instability, and shortages of trained specialists continue to hinder timely diagnosis and access to evidence-based interventions. 12 13 Recommended therapies—such as behavioral and communication interventions for ASD—are often excluded from insurance coverage, creating additional barriers for families with fewer socioeconomic resources. Available epidemiologic studies are limited in number and frequently rely on heterogeneous methods or subpopulation-based samples. The prevalence of ADHD, for example, has been estimated at 5.8% among Brazilian adolescents aged 12 to 14 years 14 and at 10.5% among individuals younger than 19 years of age in Chile. 15 The reported ASD prevalence also varies widely, with estimates of 0.17% among Venezuelan children aged 3 to 9 years 16 and of 0.11% among Ecuadorian children aged 5 to 15 years, 17 both below the approximate 1% pooled global estimate. 18 For intellectual disability, an Argentinian study 19 reported a prevalence of 0.93% for all forms (IDID: 0.66%) among individuals younger than 19 years of age. However, these studies lack longitudinal or age-standardized estimates, limiting assessment of temporal changes and broader epidemiological patterns in the region.

Global GBD analyses 20 21 22 suggest heterogeneous long-term patterns across NDDs worldwide, with age-standardized ASD prevalence tending to rise modestly, ADHD showing slight declines, and IDID decreasing in most world regions. Regional analyses for South America using the most recent data have not yet been conducted. To address this gap, the present study uses the newly-released GBD 2023 dataset 23 to evaluate country-specific trends until 2023. Sex-specific ASPR estimates and male-to-female ratios (MFRs) are incorporated to characterize long-term sex disparities across NDD ASPRs, enabling a more demographically-detailed assessment than was previously available for the region.

## METHODS

### Data sources

The main data source was the GBD 2023 database. 23 For each disorder, sequential systematic reviews were conducted in major electronic databases (PsycINFO, Embase, and PubMed), complemented by gray literature and expert input. Case definitions followed established diagnostic criteria from the Diagnostic and Statistical Manual of Mental Disorders, third edition (DSM-III) to the text revision of the fifth edition 5 (DSM-5-TR) and from the International Classification of Diseases, ninth revision (ICD-9) to the eleventh revision (ICD-11). Although the broader GBD framework incorporates data coded using the Chinese Classification of Mental Disorders, this classification contributes no primary data from South America; therefore, it does not affect the estimates herein used.

A methodological refinement introduced in GBD 2021 and retained in GBD 2023 concerned ASD: ASPR-relevant inputs derived from passive case-finding sources (such as administrative databases, registries) were excluded because of systematic underestimation. 6 7 23 The GBD 2023 23 also incorporated additional population-based studies published since the previous iteration, expanding the evidence base for ASD. For ADHD and IDID, the definitional criteria were unchanged, but newly-available studies 6 7 were added to the nonfatal estimation pipeline.

Nonfatal estimation followed standardized procedures, using meta-regression with Bayesian regularization and trimming (MR-BRT) for empirical bias adjustment. Disease Modeling Meta-Regression, version 2.1 (DisMod-MR 2.1, Institute for Health Metrics and Evaluation [IHME]), was used for Bayesian disease modeling and to ensure internal consistency across epidemiological parameters. 6 24 Modeling decisions, including covariate selection and exclusion of outlying data points, were determined centrally by the GBD research team. Estimation adhered to the Guidelines for Accurate and Transparent Health Estimates Reporting (GATHER). 25

### Measures

The ASPRs (%) and the corresponding 95% uncertainty intervals (95%UIs) for each disorder, country, and year from 1990 through 2023 were obtained from the Global Health Data Exchange (GHDx; https://ghdx.healthdata.org/ ). The population denominators used in GBD estimation derive from multiple censuses and vital registration systems worldwide. 6 Sex-specific ASPRs were extracted to compute MFRs and examine long-term sex disparities.

### Data analyses

Temporal trends were assessed using the average annual percent change (AAPC) derived from log-linear regression, a standard approach in epidemiological trend analysis. 26 The AAPC was calculated as [exp(β) − 1] × 100, where β is the slope of the regression of the natural logarithm of ASPR on calendar year, with year centered at its mean to improve model stability. The corresponding 95%UIs were derived from the standard error of β.

The MFRs were computed annually as the male ASPR divided by the female ASPR for each disorder and country. Long-term sex-disparity trends were summarized using linear-regression slopes fitted to annual MFR values, representing the average annual change in sex disparity.

Long-term changes in ASPRs were quantified using relative change (RC%) between 1990 and 2023, based on GBD point estimates. The RC% values were interpreted as increases or decreases when the corresponding 95%UI did not include zero. All analyses were performed using the R software (R Foundation for Statistical Computing), version 4.4.5. Statistical significance was defined as two-sided *p* < 0.05.

## RESULTS

### Overall ASPRs in 1990 and 2023

Country-specific ASPRs for 1990 and 2023 are shown in Table 1 . In 2023, the ASD ASPRs ranged from 0.57% in Guyana to 1.06% in Chile; The ADHD ASPRs ranged from 1.00% in Colombia to 2.55% in Suriname; and the IDID ASPRs ranged from 0.42% in Colombia to 0.54% in Uruguay. According to the GBD 2023 95%UIs, most country-level changes between 1990 and 2023 for ASD and ADHD were small and often statistically uncertain, whereas IDID showed consistent declines in all countries. The relative cross-country ranking of ASPRs in 2023 was similar to those of earlier years, with Chile, Suriname, and Uruguay exhibiting the highest ASPRs for ASD, ADHD, and IDID respectively.

**Table 1:** Age-standardized prevalence rates of neurodevelopmental disorders in 12 South American countries in 1990 and 2023 (GBD 2023), with relative change and average annual percent change

NDD	ASPR 1990 (%)	ASPR 2023 (%)	RC% (95%UI)	AAPC (95%UI)
**Argentina**				
ASD	0.86 (0.39–1.76)	1.04 (0.56–1.75)	21 (−35 to 161)	0.58 (0.56–0.60)
ADHD	1.03 (0.72–1.42)	1.01 (0.72–1.42)	−2 (−14 to 15)	−0.10 (−0.13 to −0.07)
IDID	0.62 (0.20–1.26)	0.53 (0.18–1.09)	−15 (−45 to 28)	−0.49 (−0.54 to −0.45)
**Bolivia**				
ASD	0.53 (0.25–1.08)	0.64 (0.33–1.12)	21 (−37 to 151)	0.60 (0.58–0.61)
ADHD	1.72 (1.23–2.37)	1.76 (1.27–2.43)	3 (2–3)	0.09 (0.08–0.09)
IDID	0.64 (0.30–1.14)	0.51 (0.22–1.03)	−20 (−52 to 12)	−0.79 (−0.81 to −0.76)
**Brazil**				
ASD	0.49 (0.21–1.07)	0.58 (0.29–1.04)	19 (−38 to 164)	0.54 (0.51–0.57)
ADHD	1.82 (1.27–2.52)	1.93 (1.31–2.72)	6 (−4 to 16)	0.39 (0.28–0.51)
IDID	0.56 (0.24–1.07)	0.49 (0.22–0.94)	−13 (−39 to 18)	−0.46 (−0.47 to −0.44)
**Chile**				
ASD	0.83 (0.37–1.73)	1.06 (0.57–1.81)	28 (−31 to 178)	0.79 (0.76–0.81)
ADHD	1.02 (0.72–1.40)	1.05 (0.74–1.44)	3 (2–5)	0.10 (0.09–0.10)
IDID	0.59 (0.19–1.23)	0.50 (0.17–1.05)	−16 (−46 to 32)	−0.50 (−0.53 to −0.46)
**Colombia**				
ASD	0.61 (0.29–1.22)	0.76 (0.41–1.31)	25 (−31 to 159)	0.72 (0.70–0.74)
ADHD	0.98 (0.67–1.38)	1.00 (0.70–1.38)	2 (−10 to 17)	0.01 (−0.03 to 0.05)
IDID	0.51 (0.19–1.02)	0.42 (0.15–0.87)	−17 (−49 to 32)	−0.63 (−0.69 to −0.58)
**Ecuador**				
ASD	0.56 (0.26–1.14)	0.66 (0.34–1.18)	18 (−37 to 141)	0.53 (0.51–0.54)
ADHD	1.71 (1.23–2.37)	1.75 (1.26–2.42)	2 (2–3)	0.09 (0.08–0.09)
IDID	0.58 (0.25–1.11)	0.49 (0.19–0.98)	−17 (−48 to 18)	−0.58 (−0.59 to −0.56)
**Guyana**				
ASD	0.48 (0.22–1.00)	0.57 (0.29–1.00)	18 (−40 to 148)	0.51 (0.50–0.53)
ADHD	2.47 (1.79–3.43)	2.54 (1.83–3.52)	3 (2–3)	0.08 (0.07–0.09)
IDID	0.52 (0.22–0.99)	0.46 (0.20–0.91)	−11 (−38 to 15)	−0.31 (−0.33 to −0.28)
**Paraguay**				
ASD	0.53 (0.25–1.09)	0.59 (0.31–1.02)	12 (−39 to 131)	0.35 (0.35–0.36)
ADHD	1.77 (1.24–2.43)	1.80 (1.26–2.46)	1 (1 to 2)	0.05 (0.04–0.06)
IDID	0.47 (0.20–0.91)	0.45 (0.18–0.89)	−4 (−27 to 37)	−0.08 (−0.12 to −0.04)
**Peru**				
ASD	0.56 (0.26–1.15)	0.67 (0.34–1.20)	20 (−35 to 147)	0.59 (0.57–0.61)
ADHD	1.71 (1.23–2.37)	1.74 (1.25–2.40)	2 (1–2)	0.05 (0.05–0.05)
IDID	0.53 (0.22–1.02)	0.45 (0.17–0.90)	−16 (−45 to 17)	−0.56 (−0.58 to −0.54)
**Suriname**				
ASD	0.49 (0.23–1.02)	0.59 (0.30–1.03)	20 (−38 to 154)	0.59 (0.57–0.61)
ADHD	2.52 (1.82–3.49)	2.55 (1.83–3.52)	2 (1–2)	0.05 (0.05–0.06)
IDID	0.50 (0.19–0.99)	0.46 (0.19–0.92)	−8 (−34 to 23)	−0.30 (−0.32 to −0.28)
**Uruguay**				
ASD	0.89 (0.40–1.83)	1.06 (0.57–1.80)	19 (−35 to 160)	0.57 (0.55–0.59)
ADHD	1.03 (0.73–1.43)	1.05 (0.74–1.44)	1 (0–2)	0.05 (0.04–0.05)
IDID	0.63 (0.20–1.28)	0.54 (0.19–1.11)	−13 (−44 to 51)	−0.43 (−0.44 to −0.41)
**Venezuela**				
ASD	0.61 (0.29–1.22)	0.73 (0.39–1.23)	19 (−35 to 145)	0.59 (0.57–0.62)
ADHD	2.07 (1.49–2.83)	2.08 (1.49–2.83)	0 (−1 to 1)	−0.03 (−0.08 to 0.01)
IDID	0.50 (0.19–0.96)	0.44 (0.15–0.92)	−11 (−43 to 20)	−0.34 (−0.40 to −0.28)

Abbreviations: 95%UI, 95% uncertainty interval; AAPC, average annual percent change; ADHD, attention deficit/hyperactivity disorder; ASD, autism spectrum disorder; ASPR, age-standardized prevalence rate; IDID, idiopathic developmental intellectual disability; GBD 2023, Global Burden of Disease Study 2023; NDD, neurodevelopmental disorder; RC%, relative change.

Notes: The AAPCs were estimated from log-linear regression of yearly ASPRs. Positive RC% or AAPC values indicate an increasing trend; negative values indicate a decline. The estimates correspond to both sexes combined, exclude regional or global aggregates, and are based on GBD 2023 point estimates.

### Long-term relative changes (1990–2023)

The RC% values indicated modest increases in the ASD ASPRs in all countries, with estimates ranging from +0.12% in Paraguay to +0.28% in Chile. The ADHD ASPRs showed little net change, with RC% values ranging from −0.02% in Argentina to +0.06% in Brazil. In contrast, the IDID ASPRs declined in every country, with RC% estimates ranging from −0.04% in Paraguay to −0.20% in Bolivia. Several IDID UIs excluded zero, indicating statistically-robust declines in multiple countries ( Table 1 ).

### Temporal trends (1990–2023)

**Figure 1:**
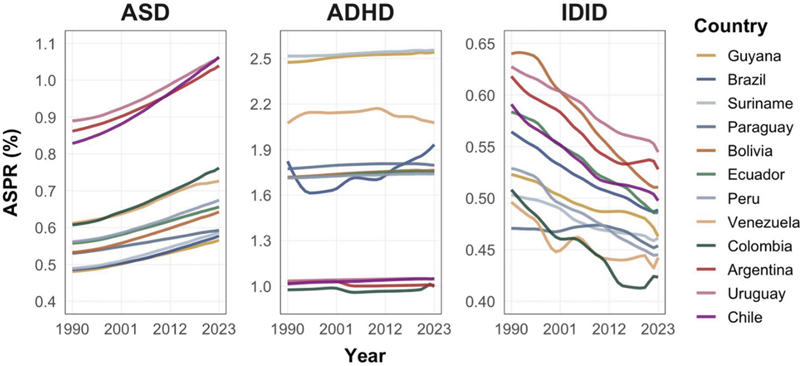
Age-standardized prevalence rate (ASPR) trajectories for autism spectrum disorder (ASD), attention deficit/hyperactivity disorder (ADHD), and idiopathic developmental intellectual disability (IDID) in South America, 1990–2023. The panels display ASD ( **A** ), ADHD ( **B** ), and IDID ( **C** ). The solid lines represent country-specific ASPRs (%) for both sexes from 1990 to 2023. Countries included: Argentina, Bolivia, Brazil, Chile, Colombia, Ecuador, Guyana, Paraguay, Peru, Suriname, Uruguay, and Venezuela. The Y-axis scales differ across panels to optimize visualization of disorder-specific variability. The estimates are from the Global Burden of Disease Study 2023 (GBD 2023).

**Figure 2:**
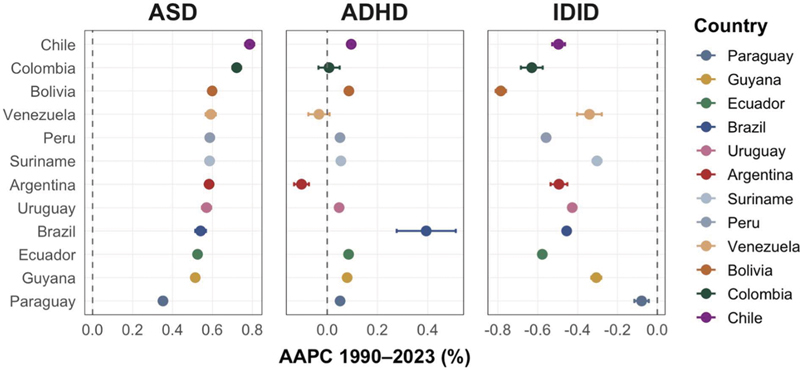
Average annual percent change (AAPC) in ASPRs for ASD, ADHD, and IDID in South America, 1990–2023. The forest plots present country-specific AAPC estimates (% per year) with 95% uncertainty intervals (95%UIs), derived from log-linear regression of annual ASPRs. The countries are ordered by AAPC magnitude within each disorder. The vertical dashed line indicates AAPC = 0. The estimates are from the GBD 2023.

Annual trajectories are presented in Figure 1 . The examination of country-specific AAPCs ( Table 1 ) showed that the ASD ASPRs increased in all 12 countries, with AAPCs from +0.35%/year in Paraguay to +0.79%/year in Chile. The ADHD ASPR trends showed minimal overall change, with AAPCs from −0.10%/year in Argentina to +0.39%/year in Brazil. The IDID ASPRs declined steadily across all countries, with AAPCs from −0.79%/year in Bolivia to −0.30%/year in Suriname. Taken together, these ranges indicate gradual increases in ASD ASPRs, broadly stable ADHD ASPRs, and uniform reductions in IDID ASPRs across national estimates. Cross-country variability in slopes was more pronounced for IDID than for ASD or ADHD, which is consistent with the heterogeneity observed in Figure 2 .

### Sex-specific ASPR patterns

Sex-stratified ASPRs ( **Supplementary Material Tables S1** and **S2** –available at https://www.arquivosdeneuropsiquiatria.org/wp-content/uploads/2026/02/ANP-2025.0295-Supplementary-Material-Table-S1.docx ) confirmed higher ASPRs in males than in females for ASD and ADHD across all 12 countries, while IDID showed minimal sex differences. In 2023, the male ASD ASPRs ranged from 0.72 to 1.30%, compared with 0.43 to 0.82% among female individuals. The male ADHD ASPRs ranged from 1.24 to 3.15%, whereas the female ADHD ASPRs ranged from 0.75 to 2.00%. The IDID ASPRs showed narrow sex gaps, ranging from 0.44 to 0.56% in male individuals and from 0.40 to 0.52% in female individuals.

**Figure 3:**
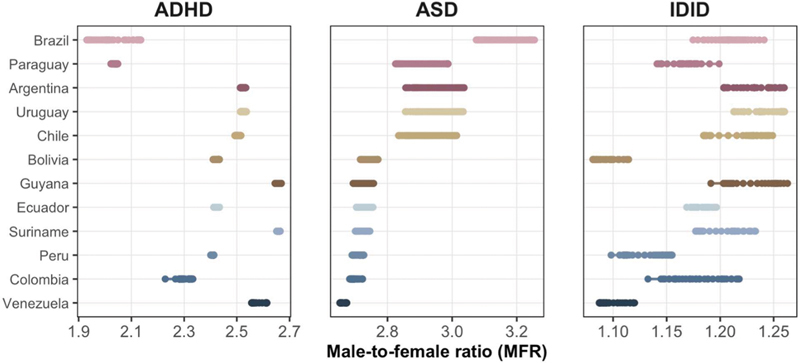
Male-to-female ratio (MFR) in ASPRs for ASD, ADHD, and IDID across 12 South American countries, 1990–2023. Each point represents the annual MFR (male ASPR divided by female ASPR) for a given country and year. The countries are ordered according to their ASD MFR in 2023, and this order is retained across all panels to facilitate cross-disorder comparisons. ASD shows the largest sex disparities, with MFRs typically ranging from approximately 2.5 to > 3.0 and relatively-stable trajectories over time. ADHD shows similar male predominance, but with lower absolute ratios and limited temporal variability. IDID shows smaller sex differences, with MFRs concentrated between 1.05 and 1.25. The values are based on sex-specific ASPRs from the GBD 2023 dataset.

The annual MFRs, shown in Figure 3 , displayed a slight upward trajectory for ASD in most countries, suggesting a gradual widening of sex disparities over time. The ADHD MFRs remained largely stable, and the IDID MFRs showed minimal year-to-year variation. The long-term MFR slopes ( **Supplementary Material Table S2** –available at https://www.arquivosdeneuropsiquiatria.org/wp-content/uploads/2026/02/ANP-2025.0295-Supplementary-Material-Table-S2.docx ) ranged from −0.0056 to +0.0058 for ASD, from −0.0062 to +0.0017 for ADHD, and from −0.0021 to −0.0005 for IDID, which is consistent with greater sex differentiation in ASD and little sex-related variation in IDID.

## DISCUSSION

The current study used GBD 2023 estimates to describe ASPRs and long-term trends for ASD, ADHD, and IDID in 12 South American countries from 1990 to 2023. By combining country-specific ASPRs, AAPCs, RC%, and sex-specific measures, it provides a regional overview of how these neurodevelopmental disorders have evolved over more than 3 decades.

The ASD ASPRs increased modestly in all countries. Compared with reports from high-income settings, 20 27 where larger relative increases have been documented over similar periods, the changes herein observed were smaller. This pattern is consistent with persistent diagnostic constraints in the region, including a limited number of trained specialists, uneven access to standardized screening and diagnostic services, and broader health-system inequities. 12 13 28 Diagnostic capacity remains concentrated in major urban centers, which likely contributes to systematic underascertainment among rural and socioeconomically-disadvantaged populations. 29 In such contexts, broader diagnostic criteria and greater awareness may have had a muted effect on the ASD prevalence observed.

The ADHD ASPRs were largely stable across countries, in contrast to the slight global declines reported in recent GBD-based analyses. 21 Diagnostic practices may not have changed in a uniform way across South America, and differences between school-based screening and clinic-based evaluations, 30 together with documented underrecognition in underserved populations, 31 may obscure underlying shifts in ADHD risk. Heterogeneity in how children are identified, referred, and followed could counterbalance temporal changes in exposure to developmental risk factors, resulting in broadly-stable ASPRs over time.

The IDID ASPRs declined consistently in all 12 countries, mirroring global reductions reported in GBD 2019 analyses. 22 These declines are compatible with sustained improvements in maternal, perinatal, and neonatal care across the region. Throughout recent decades, many South American countries have increased skilled birth attendance and institutional delivery, strengthened obstetric and newborn care, and expanded coverage of essential maternal–child health interventions. 32 33 Such changes are expected to reduce exposure to preventable causes of adverse neurodevelopmental outcomes, particularly those linked to complications during pregnancy, labor, and the early neonatal period. At the same time, primary epidemiological studies on IDID remain scarce in South America, limiting direct characterization of etiological patterns and increasing reliance on modeled inputs. Strengthening surveillance systems and population-based research will be important to document IDID trends and their determinants more precisely.

Environmental exposures may also contribute to the patterns observed in the current study. Prenatal exposure to air pollutants such as particulate matter with an aerodynamic diameter ≤ 2.5 μm (PM _2.5_ ) and nitrogen dioxide, as well as to heavy metals and endocrine-disrupting chemicals, has been associated 8 9 10 34 35 with higher ASD risk and with reductions in cognitive performance. Levels of industrial activity, mining, agricultural pesticide use, and urban air pollution differ across South American countries, creating distinct exposure profiles that may influence neurodevelopmental outcomes. 36 These exposures likely interact with genetic susceptibility and with the quality of prenatal and early childhood care, shaping the vulnerability to NDDs and the longer-term prevalence trends.

Structural inequities remain an important barrier to the timely detection of NDDs in the region. Health systems are still characterized by segmentation, unequal distribution of specialists, and substantial out-of-pocket costs. 12 37 38 Families with fewer socioeconomic resources often face longer travel distances, extended waiting times, and fragmented care pathways, which can delay evaluation and limit access to interventions. 39 Qualitative studies 40 from several Latin American countries describe limited coordination between the health and education sectors, a lack of culturally-adapted assessment tools, and prolonged diagnostic delays. These factors likely contribute to underascertainment, particularly for ASD and ADHD, and they may help explain the modest or minimal ASPR changes observed over time despite increased global awareness.

The sex-specific analyses provide additional insight into detection patterns. In male individuals, ASD and ADHD remained more commonly identified across all countries, and a slight widening of the ASD MFR was observed over the study period. This finding is consistent with evidence 11 38 that girls may present with subtler or more compensatory social-communication profiles, as well as different behavioral manifestations, which can reduce referral and diagnosis rates. In contrast, the ADHD MFRs were more stable, and IDID showed minimal sex differences, suggesting more comparable detection across sexes for that condition.

A key strength of the present study is the use of the GBD 2023, which incorporates updated systematic reviews, analytic refinements, and expanded data sources, including sex-specific prevalence estimates. The combined use of ASPRs, AAPCs, RC%, and MFR trajectories enabled a consistent description of long-term patterns across multiple countries. Several limitations should also be noted. First, the analysis relies on modeled estimates in settings with limited primary data, and variation in diagnostic infrastructure and service availability may influence the underlying inputs, particularly for ASD and ADHD. Second, the study focuses on prevalence rather than incidence, and age-specific trends were not examined, which limits inferences about changes in early identification and cohort effects. Third, joinpoint regression was not applied because the aim was to summarize broad, long-term patterns rather than identify specific years in which trends changed direction. In this context, the AAPC from log-linear regression offered a straightforward way to summarize the overall direction and magnitude of country-specific trends across the full observation period.

In conclusion, country-specific patterns across South America showed sustained reductions in IDID ASPRs that were not matched by comparable changes in ASD or ADHD. While IDID declined in all 12 countries, ASD showed only modest increases and ADHD remained broadly-stable over the study period. These trajectories suggest that improvements in perinatal and early-childhood health may have been more effective in preventing some causes of intellectual disability than in promoting timely identification of other neurodevelopmental conditions. The persistence of marked sex differences—particularly for ASD, in which the MFRs remained high and, in some settings, widened—further indicates that important groups, especially girls and women, may still be underrecognized or diagnosed late.

Strengthening the position of NDDs within national child and adolescent health agendas in South America will be important. Enhancing developmental surveillance, expanding training for frontline professionals, and integrating standardized assessment pathways into primary care and school-linked services could help narrow the existing detection gaps. At the same time, reliance on modeled estimates highlights the need to improve the local epidemiologic capacity and generate robust population-based data to complement and refine the GBD projections. Future research should examine how specific policy reforms, changes in service delivery, and environmental and social determinants relate to the ASPR trends herein described, to support strategies that promote earlier identification and more equitable access to care for neurodivergent populations in the region.
